# Research ethics review at University Eduardo Mondlane (UEM)/Maputo Central Hospital, Mozambique (2013–2016): a descriptive analysis of the start-up of a new research ethics committee (REC)

**DOI:** 10.1186/s12910-018-0291-4

**Published:** 2018-05-23

**Authors:** Jahit Sacarlal, Vasco Muchanga, Carlos Mabutana, Matilde Mabui, Arlete Mariamo, Assa Júlio Cuamba, Leida Artur Fumo, Jacinta Silveira, Elizabeth Heitman, Troy D. Moon

**Affiliations:** 1 Faculty of Medicine, University Eduardo Mondlane, Maputo, Mozambique; 2 Training Institute for Professors, Matola, Mozambique; 3 Maputo Central Hospital, Maputo, Mozambique; 4 University of Texas Southwestern Medical Center, Dallas, TX USA; 5 Vanderbilt University Medical Center, Nashville, TN USA

**Keywords:** Ethics, Research ethics review, Research ethics committee, Low-and middle-income country, Mozambique

## Abstract

**Background:**

Mozambique has seen remarkable growth in biomedical research over the last decade. To meet a growing need, the National Committee for Bioethics in Health of Mozambique (CNBS) encouraged the development of ethical review processes at institutions that regularly conduct medical and social science research. In 2012, the Faculty of Medicine (FM) of University Eduardo Mondlane (UEM) and the Maputo Central Hospital (MCH) established a joint Institutional Committee on Bioethics for Health (CIBS FM & MCH). This study examines the experience of the first 4 years of the CIBS FM & MCH.

**Methods:**

This study provides a descriptive, retrospective analysis of research protocols submitted to and approved by the CIBS FM & MCH between March 1, 2013 and December 31, 2016, together with an analysis of the Committee’s respective reviews and actions.

**Results:**

A total of 356 protocols were submitted for review during the period under analysis, with 309 protocols approved. Sixty-four percent were submitted by students, faculty, and researchers from UEM, mainly related to Master’s degree research (42%). Descriptive cross-sectional studies were the most frequently reviewed research (61%). The majority were prospective (71%) and used quantitative methodologies (51%). The Departments of Internal Medicine at MCH and Community Health at the FM submitted the most protocols from their respective institutions, with 38 and 53% respectively. The CIBS’s average time to final approval for all protocols was 56 days, rising to 161 for the 40 protocols that required subsequent national-level review by the CNBS.

**Conclusions:**

Our results show that over its first 4 years, the CIBS FM & MCH has been successful in managing a constant demand for protocol review and that several broad quality improvement initiatives, such as investigator mentoring and an electronic protocol submission platform have improved efficiency in the review process and the overall quality of the protocols submitted. Beyond Maputo, long-term investments in training and ethical capacity building for CIBS across the country continue to be needed, as Mozambique develops greater capacity for research and makes progress toward improving the health of all its citizens.

## Background

The need for official ethics committees to evaluate protocols for human subjects research has been well described in the Declaration of Helsinki [1], the International Ethical Guidelines for Biomedical Research in Human Subjects (CIOMS) [2], and in the regulations of the World Health Organization (WHO) [3] as an essential means to ensure the ethical acceptability of research protocols and to safeguard the dignity, rights, safety, and well-being of study participants. The major objective of the ethical evaluation of research protocols is to ensure that they meet three fundamental principles: respect for persons, beneficence, and justice in the conduct of research [4].

In Mozambique, bioethical considerations for research activities involving human beings were formally outlined in 2002 in regulations established by the National Committee for Bioethics in Health of Mozambique (*Comité Nacional de Bioética para Saud*e, CNBS), approved by the Ministry of Health (MISAU) [5], and by the General Regulations for Institutional Bioethics Committees, in force since 2011 [6]. Additional ethical considerations for research are detailed in the Mozambican Code of Ethics for Science and Technology [7] and the Code of Ethics and Deontology of the Order of Physicians of Mozambique [8].

The CNBS (FWA00003139) was initially established in 2002, in line with international efforts to protect participants in biomedical research through formal evaluation of the ethical aspects of research protocols involving human subjects. Over its first decade, the number of submitted protocols grew exponentially, particularly for clinical trials on the treatment and prevention of infectious diseases [9]. This growth was accompanied by the expansion of postgraduate degree programs in the biomedical and behavioral sciences that required candidates to conduct research. By 2010, the demands placed on the CNBS to review this growing number of protocols became unsustainable. Like many other new national research ethics committees (RECs) in recent years [10–13], the CNBS determined that international best practices and national regulations for human subjects research were sufficiently well recognized that protocols for certain kinds of research could be reviewed appropriately by authorities in the institutions from which the research originated, freeing the CNBS to concentrate on more complex and higher-risk protocols.

In 2011, the CNBS developed policies by which the nationally-required ethics review process could be conducted at the institutional level and through which institutions could create and operate their own Institutional Bioethics Committees for Health (*Comités Institucionais de Bioética para Saude*, CIBS), under the umbrella and federal wide assurance (FWA) number of the CNBS [7–10, 14]. Under the regulations established by the CNBS, a CIBS would be authorized to evaluate and approve protocols for observational epidemiological studies, qualitative studies, and monitoring from Mozambican or international researchers. Protocols related to clinical trials, those that involve the collection of biological samples, and those that involve vulnerable populations may be reviewed by an approved CIBS in order to ensure appropriate institutional oversight and follow-up, but then must be forwarded to the CNBS for final review and approval.

**Fig. 1:**
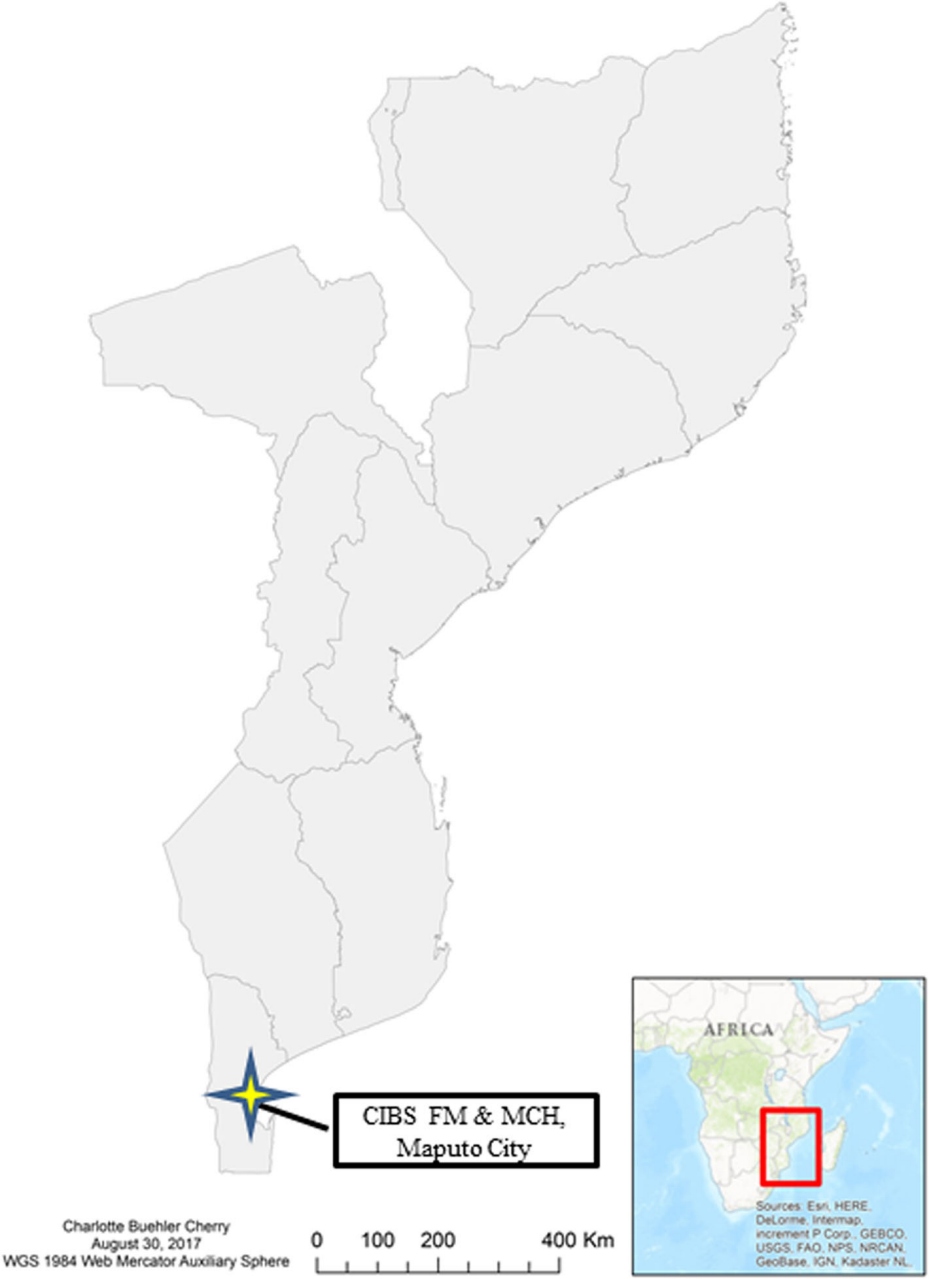
Map of Mozambique with location of the *Comité Institucional de Bioética para Saúde* (CIBS) FM & MCH and Maputo City

One of the first CIBS authorized under the new policy was a joint effort of the Faculty of Medicine (FM) of Eduardo Mondlane University (UEM) and the Maputo Central Hospital (MCH), located adjacent to the UEM campus (Fig. 1). UEM, Mozambique’s premier medical training institution, has the most evolved and productive biomedical research enterprise of the country’s academic universities and has been one of the principal points of entry for most international research collaborations conducted in the country [15]. The CNBS authorized the establishment of the joint CIBS of the FM and MCH on November 29, 2012. Since that time, CNBS has approved another six CIBSs operating at different institutions around the country [9].

The CIBS FM & MCH has 15 members, including professionals from the health, biological, social sciences and humanities; a lawyer; and a lay member who represents the interests of the community. The main responsibility of the CIBS FM & MCH is to ensure a competent and independent review of all ethical and methodological aspects of research protocols submitted by investigators from these two institutions, prior to their implementation. Upon joining the CIBS, members sign a declaration of no conflict of interest and receive training on the following topics: Ethics in Medical Research, Essentials of Good Clinical Practice for Health Professionals, and How to evaluate a research protocol.

**Fig. 2:**
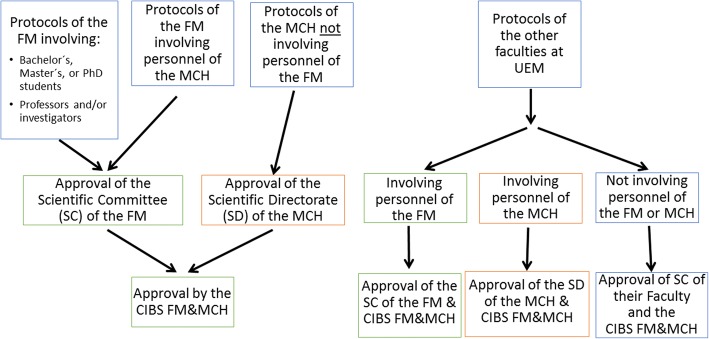
Flow of Protocols through the CIBS FM & MCH. FM = Faculty of Medicine, MCH = Maputo Central Hospital, UEM = University Eduardo Mondlane, CIBS = Comite Institucional de Bioetica para Saude

The CIBS FM & MCH meets regularly at the UEM Faculty of Medicine on the first Thursday of each month to review submitted protocols and decide on their acceptability. The flow of submitted protocols through the CIBS is slightly different for each of the two institutions, as seen in the flowchart presented in Fig. 2. In general, prior to review by the CIBS FM & MCH, protocols submitted from the FM must first be reviewed and approved by the Faculty’s Scientific Committee (SC), whereas protocols from the MCH must first be evaluated by the hospital’s Scientific Directorate (SD). After its review, the CIBS FM & MCH produces a written summary of each protocol’s evaluation, which is sent to the investigators within 15 days of the meeting.

The objective of this work is to describe the CIBS FM & MCH’s first 4 years with regards to the nature of protocols submitted for approval and its experience with the review process. Specifically, we examine: the departments from which protocols were submitted, the types of research protocols submitted, the research topic areas of greatest frequency, the average time taken to complete a review, and the most frequent causes of delay in protocol approval.

## Methods

This study was conducted by members of the CIBS FM & MCH, employing a descriptive, retrospective, and quantitative approach. Our analysis focused on data found within the CIBS FM & MCH’s paper files and electronic database, and the content of each individual research protocol submitted for review between March 2013 and December 2016. We counted overall proposals, looking at which departments they came from and measuring the time periods involved in the different stages of the review cycle. Protocols that were initially reviewed by the CIBS FM & MCH and subsequently transferred to the CNBS were included in this analysis in order to provide a comprehensive description of the types and number of research protocols submitted, and their relative length of time until approval.

For descriptive analysis, central tendency measurements (arithmetic mean and standard deviation) were used to calculate the mean time between different steps in the review process, starting with the initial submission of the protocol to the CIBS FM & MCH and ending with the sending of a letter of approval to the investigators. Chi-Square tests were used to determine associations between the study’s different variables. Data analysis was conducted using STATA, version 13.0 (StataCorp 2013, Stata Statistical Software: Release 13. College Station, TX: StataCorp LP).

## Results

### Profile of protocols evaluated

In the period from March 14, 2013 to December 31, 2016, 356 human subject research protocols were submitted to the CIBS FM & MCH for review. Of these, 309 (87%) protocols were reviewed during this same period, qualifying them for inclusion in our analysis. The remaining 47 were excluded from the present study because they were 1) transferred to another CIBS, or 2) withdrawn by the investigator. The majority of the 309 protocols were submitted by students or investigators affiliated with UEM (64%). The number of protocols evaluated varied by year. In 2013, the CIBS FM & MCH received 60 (19%) protocols; in 2014, 97 (31%) protocols; in 2015, 88 (29%) protocols; and in 2016, 64 (21%) protocols (Table 1).

**Table 1:** Characteristics of Protocols submitted to the CIBS FM&MCH: 2013–2016

*N* = 309	2013 n(%)	2014 n(%)	2015 n(%)	2016 n(%)	Total n(%)	*p-value*
Total Protocols Evaluated	60 (20)	97 (31)	88 (28)	64 (21)	309 (100)	
Source of Protocol						
UEM	45 (75)	58 (60)	53 (60)	41 (64)	197 (64)	0.024
MCH	10 (17)	9 (9)	16 (18)	12 (19)	47 (15)	
other	5 (8)	30 (31)	19 (22)	11 (17)	65 (21)	
Academic Level						
Bachelors	7 (12)	33 (34)	17 (19)	11 (17)	68 (22)	< 0.001
Masters	19 (32)	42 (43)	38 (43)	31 (48)	130 (42)	
Doctoral	3 (5)	5 (5)	5 (6)	5 (8)	18 (6)	
Post-Doctoral	1 (1)	1 (1)	7 (8)	0 (0)	9 (3)	
Faculty	30 (50)	16 (17)	21 (24)	17 (27)	84 (27)	
Type of Study						
Descriptive cross sectional	33 (55)	65 (67)	57 (65)	35 (55)	190 (61)	0.192
Qualitative studies	14 (23)	15 (16)	14 (16)	16 (25)	62 (20)	
Observation cross sectional	5 (8)	3 (3)	9 (10)	1 (2)	18 (6)	
Clinical trial	1 (2)	1 (1)	1 (1)	3 (4)	6 (2)	
Others	7 (12)	13 (13)	7 (8)	9 (14)	33 (11)	
Temporality of study						0.088
Prospective	43 (72)	68 (70)	56 (64)	53 (83)	220 (71)	
Retrospective	16 (27)	29 (30)	32 (36)	11 (17)	88 (28)	
Both	1 (1)	0 (0)	0 (0)	0 (0)	1 (0)	
Data Collection Method						
Quantitative	30 (50)	42 (43)	54 (62)	32 (50)	158 (51)	0.187
Qualitative	25 (42)	46 (48)	24 (27)	24 (38)	118 (39)	
Mixed	5 (8)	9 (9)	10 (11)	8 (12)	32 (10)	
Discipline						
Health Sciences	29 (48)	61 (63)	60 (68)	39 (61)	189 (61)	0.118
Social Sciences	19 (32)	26 (27)	19 (22)	21 (33)	85 (28)	
Biological Sciences	12 (20)	10 (10)	9 (10)	4 (6)	35 (11)	
Sub-Discipline						
Clinical Medicine	23 (38)	57 (59)	57 (64)	35 (55)	172 (56)	0.001
Laboratory	18 (30)	10 (10)	9 (10)	7 (11)	44 (14)	
Sociology	9 (15)	9 (9)	7 (8)	7 (11)	32 (10)	
Knowledge/Attitude/Practice	2 (3)	9 (9)	5 (6)	7 (11)	23 (7)	
Health Administration	0 (0)	5 (5)	3 (3)	2 (3)	10 (3)	
Anthropology	6 (10)	0 (0)	1 (1)	2 (3)	9 (3)	
Psychiatry	0 (0)	2 (2)	0 (0)	3 (5)	5 (2)	
Pharmacy	1 (2)	1 (1)	1 (1)	0 (0)	3 (1)	
Other	1 (2)	4 (4)	5 (6)	1 (2)	11 (4)	
Location of Research						0.014
Hospital	18 (30)	45 (46)	47 (53)	32 (50)	142 (46)	
Peripheral Health Facility	6 (10)	20 (21)	13 (15)	7 (11)	46 (15)	
Community	12 (20)	12 (12)	15 (17)	9 (14)	48 (16)	
Laboratory	15 (25)	12 (12)	5 (6)	4 (6)	36 (12)	
Multicenter	3 (5)	2 (2)	4 (5)	2 (3)	11 (3)	
School	3 (5)	2 (2)	1 (1)	4 (6)	10 (3)	
other	3 (5)	4 (4)	3 (3)	6 (9)	16 (5)	
Committee Decision						
Approved by CIBS FM & MCH	50 (83)	74 (76)	61 (69)	37 (58)	222 (72)	0.002
Approved by CNBS	6 (10)	12 (12)	16 (18)	6 (9)	40 (13)	
With the Investigator^a^	3 (5)	6 (6)	9 (10)	17 (27)	35 (11)	
With the CNBS^b^	1 (2)	5 (5)	2 (2)	4 (6)	12 (4)	

^a^Not approved in the study period due to being with the investigator to respond to comments from reviewers

^b^Not approved in the study period due to being under review with the CNBS

Seventy percent (70%) of all protocols submitted during the first four-years of the CIBS FM & MCH were research protocols required as part of a Bachelor’s, Master’s or Doctoral degree program. The most common types of studies submitted were descriptive cross-sectional studies 190 (61%); qualitative research (ethnographic, grounded-theory research, and phenomenological research) 62 (20%); observation cross-sectional studies 18 (6%); and clinical trial 6 (2%). Over two-thirds of the protocols submitted were for prospective studies (71%) and half used quantitative methods (51%). The majority of protocols came from the health sciences disciplines (61%), with most related to the sub-area of ​​Clinical Medicine (56%) and Laboratory (14%). Protocols in the social sciences were also common, with “Sociology” research and “Knowledge, Attitudes and Practices (KAP) studies” representing 10 and 7% of submitted protocols respectively. One hundred and forty-two of the studies reviewed were to be performed with hospital data or patients in a hospital setting (46%), followed by 48 community-based studies (16%), and 46 studies to be conducted in a peripheral health unit (15%) (Table 1).

Upon completion of their analysis and evaluation, the CIBS FM & MCH directly approved 222 (72%) of the 309 protocols submitted. An additional 40 (13%) were submitted to the CNBS, as they were deemed to be beyond the CIBS’ competence, and subsequently were approved by the CNBS during our inclusion period.

At the time of this writing, 47 protocols (15%) were pending approval; 35 were with the investigators due to a need for additional information in response to questions raised in the initial review; and 12 were pending comments from the CNBS (Table 1).

### Distribution of protocols by Departments from MCH or UEM

During the period of analysis, MCH submitted a total of 47 protocols for review by CIBS FM & MCH. Of these, the Department of Internal Medicine submitted 18 (38%), followed by the Departments of Pediatrics and Surgery with 13 (28%) and 5 (11%) protocols respectively. Investigators at UEM, by contrast, submitted 197 protocols for review during the same period, 132 (67%) from the Faculty of Medicine, 26 (13%) from the Faculty of Education, and 23 (12%) from the Faculty of Health Sciences. Within the Faculty of Medicine, the Departments of Community Health submitted 69 (53%) protocols, followed by the Department of Microbiology with 33 (25%) (Table 2).

**Table 2:** Distribution of Protocols Submitted from different Departments to the CIBS FM&MCH: 2013–2016

*N* = 309	2013 n(%)	2014 n(%)	2015 n(%)	2016 n(%)	Total n(%)	*p-value*
Department at MCH (*n* = 47)						
Internal Medicine	5 (50)	4 (44)	5 (31)	4 (34)	18 (38)	0.443
Pediatrics	2 (20)	1 (11)	5 (31)	5 (42)	13 (28)	
Surgery	0 (0)	2 (22)	2 (13)	1 (8)	5 (11)	
Pathology	2 (20)	1 (11)	1 (6)	0 (0)	4 (9)	
Ophthalmology	0 (0)	0 (0)	2 (13)	0 (0)	2 (4)	
Obstetrics/Gynecology	1 (10)	0 (0)	0 (0)	0 (0)	1 (2)	
Oncology	0 (0)	0 (0)	1 (6)	0 (0)	1 (2)	
Orthopedics	0 (0)	0 (0)	0 (0)	1 (8)	1 (2)	
Statistics	0 (0)	0 (0)	0 (0)	1 (8)	1 (2)	
Pain Unit	0 (0)	1 (11)	0 (0)	0 (0)	1 (2)	
Schools at UEM (*n* = 197)						
Faculty of Medicine	35 (78)	39 (67)	36 (68)	22 (54)	132 (67)	0.11
Faculty of Education	0 (0)	10 (17)	5 (9)	11 (27)	26 (13)	
Faculty of Health Sciences	7 (16)	5 (9)	6 (11)	5 (12)	23 (11)	
Faculty of Social Sciences	2 (4)	1 (2)	1 (2)	2 (5)	6 (3)	
Faculty of Chemistry	0 (0)	1 (2)	3 (6)	1 (2)	6 (3)	
Faculty of Veterinary Medicine	0 (0)	2 (3)	1 (2)	0 (0)	3 (2)	
Other	1 (2)	0 (0)	1 (2)	0 (0)	1 (1)	
Department of the FM (*n* = 132)						
Community Health /MPH program	16 (46)	18 (46)	20 (51)	15 (68)	69 (53)	0.022
Microbiology/Masters FELTP^a^	6 (17)	16 (41)	6 (17)	5 (22)	33 (25)	
Mental Health/Master’s program	4 (12)	4 (10)	2 (6)	1 (5)	11 (8)	
Biochemistry	1 (3)	0 (0)	0 (0)	0 (0)	1 (1)	
Other	8 (22)	1 (3)	8 (26)	1 (5)	18 (13)	

^a^Masters FELTP = Master’s degree in Field Epidemiology and Laboratory Training Program

### Average protocol approval time

**Fig. 3:**
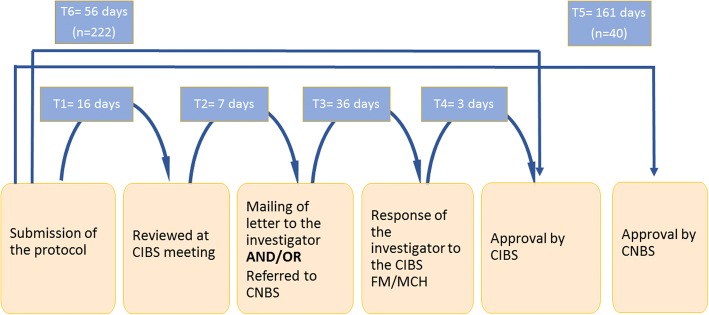
Flow Chart for Approval of Protocols and the Average Time in Days Taken at Each Step. *47 of 309 total protocols submitted were not included in the time analysis as they are still pending approval at the time of writing

The average length of time between the submission of a protocol and its final approval by the CIBS FM & MCH (*n* = 222) was 56 days (SD; 60.3; 95% CI: 48–64). For those protocols that required further evaluation by the CNBS after initial review by the CIBS (*n* = 40), the average length of time to approval increased to 161 days (SD: 82 days; 95% CI: 134–187) (Fig. 3).

The average time between a protocol’s submission to the CIBS FM & MCH and its first review at a monthly committee meeting was 16 days (SD: 11 days; range 0–76). The average time between that first review meeting and the CIBS’ subsequent communication of feedback to the investigator was an additional 7 days (SD: 6.5, range 0–28). The average time from when the Committee sent its comments to the investigator, until the investigator responded to the CIBS was 36 days (SD: 54.6; range: 0–331). Finally, after receiving an investigator’s response, it took the CIBS FM & MCH an average of 3 days (SD: 11.2; range: 0–133) to give its final approval. Of note, > 90% of protocols received, required some additional information or correction from the investigator prior to their being approved.

Further analysis of the average time to approval by discipline reveals that protocols from the Health Sciences took approximately 50% more time to be approved than those from the Social Sciences, and twice as long when compared to protocols in the area of Biological Sciences. Protocols that were submitted by investigators at a post-graduate level (mainly physicians in clinical residency) took longer to be approved (average of 140 days), compared to protocols from students pursuing a Master’s degree (108 days), and protocols from faculty (95 days).

## Discussion

The experiences described here were born out of a desire by investigators from the UEM Faculty of Medicine and Maputo Central Hospital to increase the quantity of research at these institutions undergoing ethical review; to ensure that the ethical review is competent and responsive to investigators needs by being efficient; and that investigators from these institutions learn about how ethical review enhances research practice. The need to decentralize the research ethics review process in Mozambique was mainly due to an increase in the demand for ethical review from both national and international researchers, as well as from Mozambican universities that are promoting academic research and publication [9]. Following this decision, the heads of UEM’s Faculty of Medicine and the Maputo Central Hospital determined that it would be faster and more effective to establish a joint committee to review the two institutions’ protocols under the regulations established by the CNBS. Since its inception in 2012, CIBS FM & MCH has evaluated every protocol that it has received, including those that required CNBS’s review, with the primary goal of gaining experience and learning to evaluate the more complicated protocols involving human samples and/or clinical trials.

It is not surprising that the Community Health and Microbiology departments of the FM submitted the largest numbers of protocols during our period of analysis. The majority of all research in the FM is conducted in these two departments, which support the majority of students conducting Master’s-level research through the Master’s degree in Public Health (Department of Community Health, MPH) and the Master’s in Field Epidemiology and Laboratory Training Program (Department of Microbiology, FELTP). The 11 protocols (8%) from the Department of Mental Health similarly highlight the research conducted in its Master’s in Mental Health program, and emphasizes how the presence of a graduate-level training programs can help to create awareness both of research in general and of the need for ethical review prior to implementation of a protocol. This finding is also in line with studies on RECs in other low- and middle-income countries that show that most protocols are submitted by students and investigators from within the Committee’s institution [16–19]. It is worth noting that the low number of research protocols from doctoral-level trainees reflects the current lack of doctoral programs in Mozambique. We anticipate that this situation will change in 2017 with the introduction of PhD programs in Public Health and Biological Sciences at UEM’s Faculty of Medicine.

Our data show that the average length of time to approval was greatest for those more complicated protocols involving the collection of biological samples and/or recruiting vulnerable populations such as children, adolescents, and pregnant women. These studies require the CIBS to forward the protocol to the CNBS for review. For these protocols, the largest bottleneck in the process was getting investigators to respond to the CNBS’ requests for more information and/or requests for revision due to the poor quality of the research protocol initially submitted (Fig. 3: T3). An investigator’s need to make considerable revisions to a protocol can delay the Committee’s review process and create hurdles that investigators may inappropriately blame on the Committee [20]. Our findings comport with several studies that have shown that ethical and procedural issues are the primary reasons for RECs’ non-approval of protocols after first review [18, 21, 22]. Similar to these studies, we found that many applications not approved in the first review had issues that included violations of procedures, missing information, and discrepancies between different parts of the application, as well as grammatical and spelling errors [21].

The large number of protocols reviewed each year by the CIBS FM & MCH reflects a growing awareness by its affiliated faculty and students of the importance of institutional accountability for ensuring ethical practices in research with human subjects. Moreover, the CIBS FM & MCH has had an important role in education and quality improvement through its reviews. In cases where committee members found that the initial protocol was not of sufficiently sound ethical or scientific quality, the CIBS FM & MCH appointed a member from a related discipline to work with the investigators to ensure the protocol is in keeping with the field’s best practices. The goal of these meetings was to improve the quality of the protocol and to prevent miscommunication about the Committee’s requested revisions. Although this step increased the time between submission and the CIBS’ initial response from 9 days to 14 days (Fig. 3: T1 & T2), it also reduced the subsequent time taken by researchers to resubmit their revised protocols from 50 to 39 days (Fig. 3: T3), and improved the final quality of the protocols submitted. This type of face-to-face review is similar to a process used in the United Kingdom, in which “Ethics Officers” assist with a pre-review of protocols in order to identify possible problems before the protocol is reviewed by the REC [23]. While pre-review does not necessarily lead to a shorter time-to-approval, an REC’s individualized efforts to educate its institution’s researchers about common problems and mistakes in submitted protocols can create a more open environment and reduce overall delays in the time for review [23].

Our examination of the data found a profound delay in the time to final approval for protocols classified as coming from post-graduate students and young physicians at MCH, whose protocols frequently raised methodological and ethical issues. These problems are likely due to the many demands on young clinicians’ time and their need to prepare their protocols at the same time as completing multiple clinical tasks and other hospital responsibilities. It may also be due to a lack of full awareness on their part of the requirements for their protocols’ review, insight into the time needed to develop a specialized clinical research protocol, and senior faculty members’ limited availability for oversight and mentoring.

This study’s conclusions on the reasons for researchers´ delayed responses to either the CIBS FM & MCH or CNBS (Fig. 3: T3) are limited by the lack of documented information on this question. Similarly, our conclusions are also limited on why the Committees take the time they do to respond to the researchers (Fig. 3: T2). Further exploration is warranted into these two points to determine whether and how overall time to approval can be reduced and the quality of protocols can be improved.

One of the CIBS FM & MCH’s approaches to reducing time-to-approval and increasing the Committee’s capacity to do self-assessment was the development of a computer platform that was implemented at the end 2017. This platform is based in a mainframe computer at the FM (www.cibs.uem.mz) and serves as an electronic system for both submitting and storing protocols. It provides researchers with the instructions and templates necessary to complete an application for protocol review, including: standard protocol templates, informed consent templates, regulations and guidance, and other necessary administrative documentation. We anticipate conducting an in-depth review of its use in the coming year.

## Conclusions

Our analysis indicates that there is a strong demand from researchers at UEM’s Faculty of Medicine and the Maputo Central Hospital for ethical review of their protocols. Over the past 4 years, most of the protocols submitted for review were for projects in the Health Sciences to be conducted by degree-seeking students from the Faculty of Medicine. Given that research is now required of post-graduate students in other programs with similarly-sized enrollments, this disproportionate use from the FM may reveal a lack of knowledge on the part of other disciplines about the need to follow specific ethical standards in the research they conduct, and represents an opportunity for the CIBS FM & MCH to educate faculty and students in UEM’s other disciplines and programs about the research ethics review process and the responsible conduct of research.

Since 2011, the National Committee for Bioethics in Health of Mozambique (CNBS) has approved the launching of 6 additional decentralized research ethics committees at various institutions around the country [9]. We describe here the first 4 years of the joint institutional research ethics committee of the UEM Faculty of Medicine and Maputo Central Hospital (CIBS FM & MCH). Our results show that over this time, the CIBS FM & MCH has been successful in managing a constant demand for protocol review and has introduced several broad quality improvement initiatives, such as investigator mentoring and an electronic protocol submission platform to improve efficiencies in the review process and the overall quality of the protocols submitted. As this is the first study published in the peer reviewed literature on research ethics review capacity in Mozambique, it is important to note that the success and capacity of the CIBS FM & MCH is not generalizable to all CIBS that have been approved and launched across the country. The CIBS FM & MCH benefits from location in the capital city Maputo and its proximity to the CNBS, also located in Maputo, as well as proximity to the Government Ministries of Health, Education, and Science and Technology. Additionally, UEM has a much longer institutional history for promoting research and as such benefits from a greater depth of potential resources, both human and financial, to support its functioning. Beyond Maputo, long-term investments in training and ethical capacity building for CIBS across the country continue to be needed, as Mozambique develops greater capacity for research and makes progress toward improving the health of all its citizens.

## Abbreviations

CIBS FM&MCH: Comité Institucional de Bioética para Saúde de Faculdade de Medicine e Hospital Central de Maputo
CIBS: Comité Institucional de Bioética para Saúde
CIOMS: Council for International Organizations of Medical Sciences
CNBS: Comite Nacional de Bioética para Saúde
FELTP: Field Epidemiology and Laboratory Training Program
FM: Faculty of Medicine
FWA: Federal Wide Assurance
KAP: Knowledge, Attitude, and Practice
MCH: Maputo Central Hospital
MISAU: Ministério de Saúde
MPH: Master of Public Health
REC: Research Ethics Committee
SC: Scientific Committee
SD: Scientific Directorate
UEM: University Eduardo Mondlane
WHO: World Health Organization
